# Resveratrol Ameliorates Mitochondrial Elongation via Drp1/Parkin/PINK1 Signaling in Senescent-Like Cardiomyocytes

**DOI:** 10.1155/2017/4175353

**Published:** 2017-10-22

**Authors:** Xuecong Ren, Li Chen, Jing Xie, Zhifeng Zhang, Gengting Dong, Jie Liang, Liang Liu, Hua Zhou, Pei Luo

**Affiliations:** ^1^State Key Laboratory of Quality Research in Chinese Medicine, Macau University of Science and Technology, Avenida Wai Long, Taipa, Macau; ^2^Faculty of Chinese Medicine, Macau University of Science and Technology, Taipa, Macau

## Abstract

Resveratrol is widely known for its antiaging properties and exerts cardiovascular protective effects in different experimental models. The role of resveratrol in regulating mitochondrial functions and dynamics during the cardiac aging process remains poorly understood. In this study, the effects of resveratrol on mitochondrial morphology and mitochondrial depolarization and on expressions of Drp1, parkin, PINK1, and LC3 were investigated in H9c2 cells after D-galactose treatment that induced senescent-like cardiomyocytes. The results show that downregulation of Drp1 markedly increased mitochondrial elongation. Senescent-like cardiomyocytes were more resistant to CCCP-induced mitochondrial depolarization, which was accompanied by suppressed expression of parkin, PINK1, and LC3-II. Resveratrol treatment significantly increased Drp1 expression, ameliorated mitochondrial elongation, and increased the mitochondrial translocations of parkin and PINK1. In addition, resveratrol significantly enhanced LC3-II expression and decreased TOM20-labeled mitochondrial content. Resveratrol also suppressed the phosphorylation of parkin and PINK1, which may relate to its abilities to degrade the impaired mitochondria in senescent-like cardiomyocytes. These findings show that suppressing mitochondrial elongation in a Drp1-dependent manner is involved in the effect of resveratrol on attenuating the development of aging cardiomyocytes. Activation of parkin and PINK1 may be a potential mechanism of resveratrol for treating cardiovascular complications related to aging.

## 1. Introduction

Age-related loss or attenuation of myocardial ischemic preconditioning (IPC) has been studied in animals and humans [1, 2]. Although the dysfunctional IPC mechanisms underlying the aging process remain unclear, there is considerable agreement that mitochondria play a key role in the aging process and that specific defects in mitochondrial function are associated with age-related decline in cardiac efficiency. Alterations of mediator release and/or intracellular pathways mediated by mitochondria may be responsible for the age-related IPC reduction. However, therapeutic intervention via mitochondrial-related mechanisms, such as ATP-sensitive potassium channels (K_ATP_ channels) and permeable transition pore openings [3–5], showed disappointing outcomes in aged hearts [6].

In the past decade, the role of mitochondrial dynamics focusing on organelle fission and fusion has been studied in normal and diseased hearts, and the dysfunction of mitochondrial fission and fusion was well implicated in cardiac death or disease with aging [7–9]. Cardiomyocytes are particularly vulnerable to ischemia due to their high-energy utility and no reserve. Therefore, the tolerance conferred by IPC in aged cardiomyocytes is probably dependent on their ability to maintain mitochondrial dynamism, such as fusion, fission, biogenesis, and selective degradation. The predominant molecular mediator of mitochondrial fission is a member of the dynamin family of GTPases named dynamin-related protein-1 (Drp1) which modulates mitochondrial dynamics. Mitochondrial fragmentation frequently observed in ischemic cardiomyocytes is widely recognized as evidence of increased mitochondrial fission mediated by Drp1 [10]. Pharmacological inhibition of Drp1 with the Mdivi-1 compound has been suggested to reduce cell death after myocardial infarction [11, 12]. Although a few studies have tested this approach and early results appear promising, the essential requirement of Drp1 in cardiac aging remains elusive. One major challenge in the investigation of mitochondrial fission is to discern the effects of cardiomyocyte senescence from those produced by DNA mutations, metabolism disorders, and excessive ROS (reactive oxygen species) production [13, 14], which contributed to mega, giant, or enlarged mitochondria [15, 16]. Mitochondria with these abnormal dynamics exhibited functional disorders and probably were responsible for the decreased response of aging hearts to IPC.

The selective removal of damaged mitochondria plays a crucial role in maintaining mitochondrial homeostasis maintenance and normal cellular metabolisms of cells. However, a deficiency in mitochondrial fission proteins results in increased activity of senescence-associated *β*-galactosidase and mitochondrial elongation in aging hearts [17]. Elongated mitochondria display a larger size that increases the difficulty of removal and always presents defective fission. Although whether Drp1 is essential for the selective removal of damaged mitochondria remains unclear, Drp1 is strongly expressed in heart and brain tissues compared to other tissues [18]. There is evidence indicating that cardiac-specific Drp1-knockout mice developed mitochondrial dysfunction and suppressed selective mitochondrial removal [19]. Drp1-mediated mitochondrial fission promoted parkin translocation in cardiomyocytes, which was disturbed by the inhibition of Drp1 [20]. PTEN-inducible kinase 1 (PINK1), a mitochondrial kinase, displayed outer membrane accumulation and initiated parkin translocation in the heart [7], implying that the selective removal of mitochondria in heart tissue is related to PINK1. Based on these previous studies, we hypothesized that the attenuation of abnormal mitochondrial elongation could restore the protective function of IPC in aged hearts by a new mechanism involving Drp1 and parkin.

Resveratrol, a natural polyphenol compound present in several plants, was shown to display antioxidant properties [21] and extend lifespan [22]. Resveratrol was also shown to downregulate lipid peroxidation and upregulate Mn-SOD to decrease oxidative stress in cardiovascular diseases [23]. Resveratrol was reported to restore the cardioprotective effect of IPC on aged hearts by enhancing cardiac function and reducing ischemia/reperfusion-induced cell apoptosis [24]. As a potential activator of sirtuin 1 (SIRT1), resveratrol improved cardiac function through SIRT1-mediated signaling pathways in aged hearts [25]. Moreover, SIRT1 inhibition diminished the preconditioning effect of resveratrol, demonstrating that the SIRT1 pathway was implicated in resveratrol preconditioning [26]. Additionally, several studies have reported the effects of resveratrol on the regulation of mitochondrial morphology and dynamics through Drp1-parkin-PINK1 signaling. Therefore, it is possible that Drp1-parkin-PINK1 signaling could be involved in the abnormal mitochondrial dynamics during cardiac aging and the cardioprotective effect of resveratrol.

In this study, we investigated the mitochondrial dynamic alterations in H9c2 cells in response to D-galactose induction, which was characterized by increased levels of senescence-associated *β*-galactosidase and BrdU incorporation. D-Galactose intervention generates reactive oxygen species and induces calcium overloading in cardiomyocytes, which are regarded as a potential mechanism in aging research. We explored the roles of mitochondrial depolarization and parkin/PINK1 translocation on elongated mitochondria in D-galactose-induced senescent-like H9c2 cardiomyocytes. To the best of our knowledge, this study is the first to propose a new mechanism of resveratrol, in which it modulates Drp1 to protect against cardiac aging disorders. The new roles of parkin and PINK1 activation required for the elimination of aging mitochondria and their importance in uncovering the antiaging functions of resveratrol are also discussed herein.

## 2. Materials and Methods

### 2.1. Cell Culture and Pharmacological Treatments

A rat H9c2 cardiomyocyte cell line, obtained from American Type Cultural Collection (CRL1446, ATCC, USA), was cultured in Dulbecco's modified Eagle's medium (DMEM, Gibco, Oklahoma, USA) supplemented with 10% fetal bovine serum (FBS, Gibco, Oklahoma, USA) and 1% *v*/*v* penicillin/streptomycin (Gibco, Oklahoma, USA) at 37°C in a 5% CO_2_ humidity environment. D-Galactose (D-Gal, ≥99%, Sigma, USA) was dissolved in DMEM and given for 48 hours. Carbonyl cyanide 3-chlorophenylhydrazone (CCCP, Sigma, USA) and resveratrol (RSV, purity > 98%, HPLC, Chengdu Conbon Bio-Tech Co. Ltd., Sichuan, China) were dissolved in dimethyl sulfoxide (DMSO, ACROS, USA).

### 2.2. Senescence-Associated *β*-Galactosidase Staining

Senescence-associated *β*-galactosidase staining (CST, USA) was performed according to the manufacturer's protocol. Briefly, cells in a 6-well plate were washed with PBS, fixed for 15 min at room temperature with fixative solution, and incubated for 24 hours with *β*-galactosidase staining solution in a dry incubator (absence of CO_2_). H9c2 cells were observed for the development of blue color under a microscope (100x). The percentage of positive cells was calculated by counting the blue-stained cells and total cells (as a standard) in five randomized fields.

### 2.3. Cell Viability and Mitochondrial Viability

Cell viability was determined by using the 3-(4,5-dimethylthiazol-2-yl)-2,5-diphenyltetrazolium bromide (MTT, Molecular Probes, USA) assay. An absorbance of 570/650 nm was evaluated by a Multi-Mode Detection Platform (SpectraMax Paradigm, Molecular Devices, USA). For mitochondrial viability, a mitochondrial viability assay reagent (Abcam, UK) was used according to the manufacturer's protocol. Briefly, 5 × 10^4^ cells/ml were seeded in a 96-well plate. After treatment, 100 *μ*l DMEM and 100 *μ*l diluted reagent were added to each well for 4-hour incubation at 37°C. The fluorescent intensity was evaluated at 590 nm with an excitation wavelength of 550 nm. Cell viability and mitochondrial viability were calculated as the ratio to the DMSO group (set as 1.0), respectively.

### 2.4. Evaluation of Mitochondrial Morphology

H9c2 cells were seeded into a *μ*-Slide 8-well glass bottom plate (#80826, ibidi, Germany) at a total number of 7500 per well. After treatments of D-galactose (0, 10, 20, and 40 g/l, Sigma, USA) or Mdivi-1 (40 *μ*M, Sigma, USA), the cells were incubated with 50 nM MitoView Red (GeneCopoeia, USA) at 37°C for 30 min. Then, the cells were washed with PBS for three times. Mitochondrial morphology in each group was captured using a confocal microscope (Leica TCS SP8, Germany) equipped with a 63x oil immersion objective. Red fluorescence represents the mitochondria stained by MitoView Red.

### 2.5. Transmission Electron Microscopy

H9c2 cardiomyocytes were fixed in 2.5% glutaric dialdehyde overnight at 4°C and washed with PBS for three times, then postfixed in 1% osmium tetraoxide for 1-2 hours, dehydrated in a graded series of ethanol concentrations, and embedded in Sparr resin. Sections of 50–70 nm thickness were placed on copper grids that were double-stained with uranyl acetate and lead citrate. Samples were examined with an H-7650 transmission electron microscope (Hitachi, Japan).

### 2.6. ROS and Calcium Determination

To measure the cellular ROS production and calcium concentration, an H_2_DCFDA Cellular ROS Detection Assay Kit (Molecular Probes, USA) and a Fluo-4 AM calcium indicator (Thermo Scientific, USA) were used according to the manufacturers' protocols and determined by BD FACSAria III flow cytometer (BD, USA) analysis. For ROS and calcium determination, Ex 488/Em 530 nm was used. The intensity of each group was calculated by counting 10,000 cells as representation.

### 2.7. MMP Determination

To measure the mitochondrial membrane potential (MMP), a JC-1 Mitochondrial Membrane Potential Assay Kit (Abcam, Cambridge, UK) was used according to the manufacturer's protocol. The fluorescent images of JC-1 in each group were captured using a confocal microscope by red and green fluorescence. For the quantification of JC-1 intensity, H9c2 cells were seeded in a 96-well black plate with clear bottom. Ex 488/Em 530 nm and Ex 550/Em 600 nm were used, and the MMP was calculated by the ratio of red-to-green fluorescence.

### 2.8. ATP Content Assay

The ATP level of H9c2 cells was measured by using a Luminescent ATP Detection Assay Kit (Abcam, UK) according to the manufacturer's protocol. The contents of ATP were analyzed from three independent experiments and detected by a Multi-Mode Detection Platform.

### 2.9. BrdU Incorporation Assay

A BrdU incorporation assay was used to measure the cell proliferation. H9c2 cells were seeded into a *μ*-Slide 8-well glass bottom plate at a total number of 7500 per well. After treatment, the cells were incubated with medium containing 10 *μ*M BrdU (Sigma, USA) for 24 hours. Then, cells were fixed by 70% ethanol and incubated with 2 M HCl for 30 min at room temperature. 1% BSA was used for blocking, and the cells were incubated with a BrdU primary antibody (1 : 500, Abcam, UK) overnight and with a secondary antibody (1: 250) for 2 hours at room temperature. DAPI (Invitrogen, USA) was stained in the final step. A fluorescent image was detected using a confocal microscope equipped with a 63x oil immersion objective. Blue fluorescence represents DAPI staining, and red fluorescence represents BrdU. Images were analyzed by using the manufacturer's software. The percentage of positive cells was calculated by counting the double-stained cells and total cells (as a standard) in five randomized fields.

### 2.10. Evaluation of Mitochondrial Respiration

The cellular oxygen consumption rate (OCR) was measured to determine the key parameters of mitochondrial respiration using the Seahorse Bioscience XFp Extracellular Flux Analyzer (Seahorse Bioscience, USA) containing an XFp Cell Mito Stress Test Kit according to the manufacturer's protocol. H9c2 cells were seeded into an XFp cell culture miniplate at a density of 4000 cells/80 *μ*l/well and treated with D-galactose (40 g/l). The sensor cartridge for the XFp analyzer was hydrated in a 37°C non-CO_2_ incubator a day before the experiment. For calibration, the sensor cartridge was loaded with 1.5 *μ*M oligomycin (complex V inhibitor) to port A, 2 *μ*M FCCP to port B, and 0.5 *μ*M rotenone/antimycin A (inhibitors of complex I and complex III) to port C. The cellular cultural medium was replaced by 180 *μ*l/well assay medium that was prepared by supplementing XF Base Medium with 5.5 mM glucose, 1 mM pyruvate, and 2 mM L-glutamine (adjusted to pH 7.4) and incubated at 37°C for 1 hour without CO_2_. When the calibration was completed, the calibration plate was replaced with a culture miniplate into the calibrated XFp Extracellular Flux Analyzer for the Mito Stress Test. The oxygen consumption rate was calculated to evaluate mitochondrial respiration.

### 2.11. Immunoblot Analysis

Cells were washed with iced PBS and lysed with RIPA buffer (20 mM Tris-HCl (pH 7.5), 150 mM NaCl, 1 mM Na_2_EDTA, 1 mM EGTA, 1% NP-40, 1% sodium deoxycholate, 2.5 mM sodium pyrophosphate, 1 mM beta-glycerophosphate, 1 mM Na_3_VO_4_, and 1 *μ*g/ml leupeptin, Cell Signaling Technology, USA) containing protease inhibitors (Roche, Basel, Switzerland), then stored on ice for 30 min. The cell lysate was centrifuged at 13,000 rpm at 4°C for 10 min, and the supernatant was collected to a new and clear tube. The protein concentration was determined using a Bio-Rad protein assay kit (Bio-Rad Laboratory, USA). Equal amounts of proteins were boiled and separated with 8% SDS-PAGE gels and transferred to a nitrocellulose membrane (Millipore, Germany). The membrane was blocked with 5% nonfat milk in Tris-buffer saline-Tween 20 (TBST) at room temperature for 1 hour, then incubated overnight at 4°C with primary antibodies of anti-Drp1 (1 : 500, Cell Signaling Technology, USA), anti-Mfn2 (1 : 1000, Cell Signaling Technology, USA), anti-Mfn1 (1 : 500, Abcam, UK), anti-OPA1 (1 : 500, Abcam, UK), anti-Bcl-2 (1 : 500, Cell Signaling Technology, USA), anti-Bax (1 : 500, Cell Signaling Technology, USA), anti-PINK1 (1 : 500, Novus, USA), anti-parkin (1 : 500, Abcam, UK), anti-LC3 (1 : 1000, Cell Signaling Technology, USA), and anti-TOM20 (1 : 500, Abcam, UK). Membranes were washed in TBST for three times and incubated with a secondary antibody (1 : 1000) for 1 hour at room temperature. Subsequently, the membranes were washed in TBST for three times and detected using the Odyssey Scanner (Licor, USA). Actin (1 : 10,000, Sigma, USA) was used as a loading control.

### 2.12. Phos-Tag Assay

To detect phosphorylated PINK1 and parkin proteins, 8% polyacrylamide gels containing 25 *μ*M phos-tag acrylamide (Wako Chemicals, USA) and 50 *μ*M MnCl_2_ were prepared before using. Before electrophoresis, samples were mixed with 1 mM MnCl_2_. During electrophoresis, cold running buffer was used. After electrophoresis, phos-tag acrylamide gels were washed with transfer buffer containing 1 mM EDTA for 10 min with gentle agitation and then replaced with transfer buffer without EDTA for 10 min with gentle agitation. Proteins were transferred to a nitrocellulose membrane (Millipore, Germany) and analyzed by conventional immunoblotting.

### 2.13. Immunocytochemistry Analysis

H9c2 cells were seeded into a *μ*-Slide 8-well glass bottom plate at a total number of 7500 per well. After treatment, cells were washed with PBST (0.1% Tween 20 to PBS) and fixed with 4% PFA (15 min, RT), then permeabilized with 0.1% Triton X-100 (10 min, RT). The cells were washed with PBST for 3 times, blocked with 1% BSA/PBST for 1 hour at room temperature, and incubated with a primary antibody (TOM20 1 : 50, Abcam; parkin 1 : 200, Abcam; and PINK1 1 : 200, Novus) overnight in 4°C. A secondary antibody was used in 1 : 250 in room temperature for 2 hours. A fluorescent image was detected using a confocal microscope equipped with a 63x oil immersion objective.

### 2.14. Statistical Analysis

Data were analyzed using GraphPad Prism 6.0 (GraphPad Software Inc., San Diego, CA, USA), and all results were expressed as means ± SEM. Dunnett's test of one-way ANOVA was used to analyze difference between 3 or more groups. For two-group analysis, Student's *t*-test was used. Values with *p* < 0.05 were considered statistically significant.

## 3. Results

### 3.1. Resveratrol Attenuated Drp1-Mediated Mitochondrial Elongation in Response to D-Galactose Induction in H9c2 Cells

**Figure 1:**
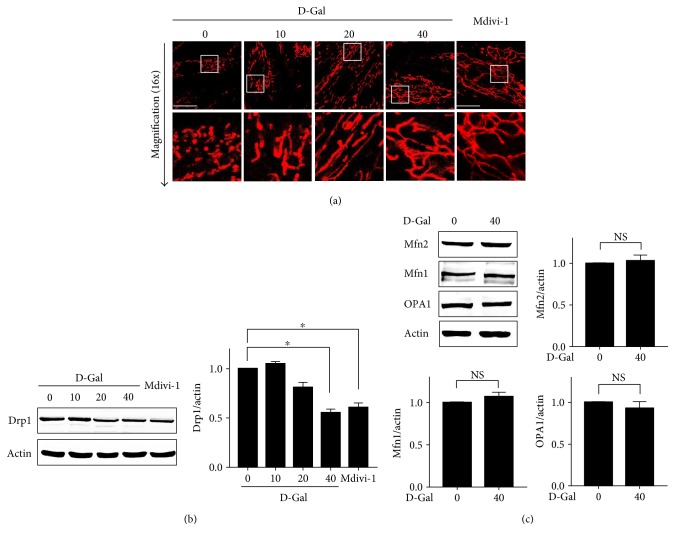
Drp1 mediated mitochondrial elongation induced by D-galactose. (a) H9c2 cardiomyocytes were treated with D-galactose (D-Gal; 0, 10, 20, and 40 g/l) and Mdivi-1 (40 *μ*M) for 48 hours, respectively. Mitochondrial morphology was detected using a confocal microscope by MitoView Red staining (upper panels); objective magnification, 63x; white scale bar represents 20 *μ*m. Magnified photographs showed a detail view of the area indicated in the upper panels. (b) Drp1 expressions in H9c2 cells treated with D-galactose and Mdivi-1 were detected by immunoblot analysis. Actin was represented as the loading control. The relative values were normalized to actin. Data (*n* = 3) were shown as the mean ± SEM (^∗^*p* < 0.05). (c) Mfn1, Mfn2, and OPA1 expressions were detected by immunoblot analysis. Actin was represented as the loading control. The relative values were normalized to actin. Data (*n* = 3) were shown as the mean ± SEM. NS: no significance (*p* > 0.05).

We examined the morphology of mitochondria in H9c2 cells exposed to D-galactose for 48 hours (Figure 1(a)). The live cell staining dye MitoView Red indicated the changes in the morphology and distribution of mitochondria after different doses of D-galactose induction. The proportion of tubular or thread-like mitochondria was significantly increased in H9c2 cells treated with D-galactose at concentrations of 10, 20, and 40 g/l. After induction with 40 g/l D-galactose, more that 80% of the mitochondria were highly elongated and completely organized into lengthy traveling chains, whereas cells without D-galactose induction displayed short or punctiform mitochondria distributed throughout their cytoplasm. Mitochondria are quite flexible and are directly correlated with the level of cardiomyocyte metabolic activity. Therefore, we wanted to investigate the mechanism underlying mitochondrial elongation or affecting mitochondrial dynamics in response to D-galactose in H9c2 cells. We evaluated fission- or fusion-regulated protein (Mfn1, Mfn2, OPA1, and Drp1) expressions, finding that Drp1 was obviously downregulated after D-galactose induction (Figure 1(b)). Compared with D0 cells (cells treated with 0 g/l D-galactose), the expression levels of Drp1 in D40 cells were significantly decreased. Conversely, there was no change in the expression levels of Mfn1, Mfn2, or OPA1 in response to D-galactose (Figure 1(c)). In addition, we further tested whether Drp1 dominated mitochondrial elongation after D-galactose induction using a selective cell-permeable Drp1 inhibitor (Mdivi-1). Mdivi-1 treatment suppressed Drp1 expression and Drp1-mediated mitochondrial fission and altered mitochondrial elongation even further in H9c2 cells, indicating that mitochondrial elongation induced by D-galactose is partially due to deficient fission machinery caused by Drp1 downregulation.

**Figure 2:**
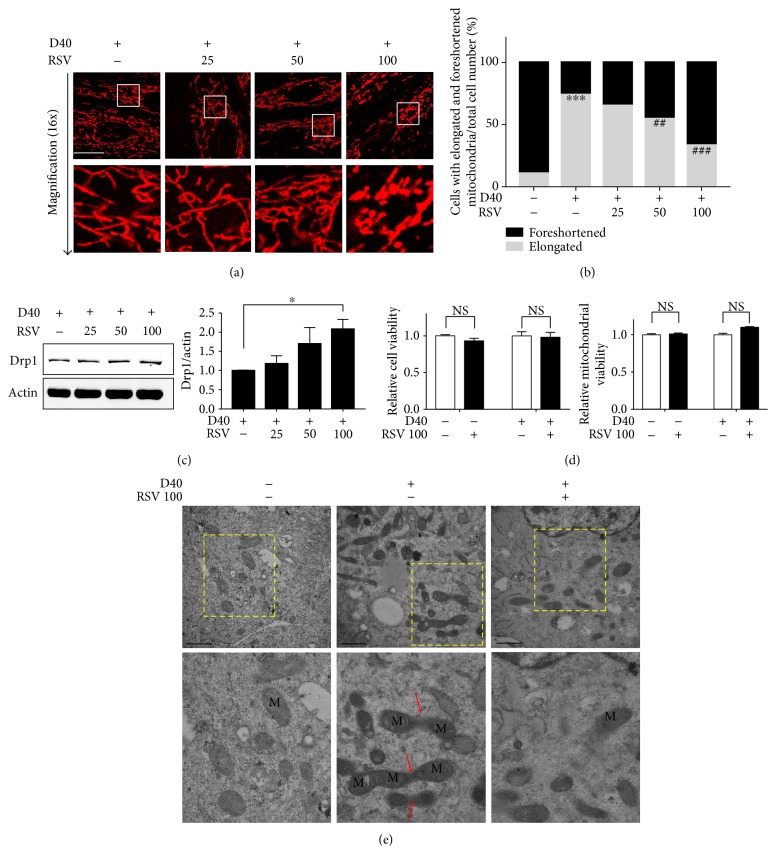
The effects of resveratrol on mitochondrial elongation and Drp1 expression. (a) H9c2 cardiomyocytes were treated with resveratrol (RSV; 25, 50, and 100 *μ*M) for 12 hours after 48-hour induction of D-Gal (40 g/l). Mitochondrial morphology was detected using a confocal microscope by MitoView Red staining (upper panels); objective magnification, 63x; scale bar represents 20 *μ*m. Magnified photographs showed a detail view of the area indicated in the upper panels. (b) Quantification of mitochondrial morphology (foreshortened and elongated) in 50 cells from fluorescent images captured by a confocal microscope. Gray bar: cells with elongated mitochondria/total cell number; black bar: cells with foreshortened mitochondria/total cell number. ^∗∗∗^*p* < 0.001 versus elongated mitochondria in cells without any treatment; ^##^*p* < 0.01 and ^###^*p* < 0.01 versus elongated mitochondria in cells treated with D-galactose (40 g/l). (c) The effect of RSV (25, 50, and 100 *μ*M) on Drp1 expressions was detected by immunoblot analysis. Actin was represented as the loading control. The relative values were normalized to actin. Data (*n* = 3) were shown as the mean ± SEM (^∗^*p* < 0.05). (d) The toxic effects of resveratrol (100 *μ*M) on H9c2 cardiomyocytes and mitochondria were evaluated. Data (*n* = 3) were shown as the mean ± SEM; NS: no significance (*p* > 0.05). (e) Electron microscopy analysis (magnification, 25,000x; upper panels) of cells treated with RSV (100 *μ*M) for 12 hours after 48-hour induction of D-Gal (40 g/l). Scale bar represents 1 *μ*m. Magnified photographs showed a detail view of the area indicated in the upper panels. The red arrow indicated the interconnected net-like mitochondria.

Next, we examined the effects of resveratrol on mitochondrial morphology in response to D-galactose. H9c2 cells were treated with D-galactose for 48 hours followed by different doses of resveratrol for 12 hours. Interestingly, live cell analysis of the changes in both mitochondrial length and distribution indicated that mitochondrial elongation was ameliorated by resveratrol in a dose-dependent manner (Figure 2(a)). In cells treated with D40 and 100 *μ*M resveratrol (RSV 100), the mitochondria packed tightly into a relatively stable phonotype and their elongation was dramatically diminished compared with that in D40 cells. Quantification of mitochondrial morphology showed that the percentage of cells exhibiting mitochondrial elongation was decreased significantly in cells treated with D40 plus 50 *μ*M or 100 *μ*M RSV cells (Figure 2(b)). We next used transmission electron microscopy to observe mitochondrial morphology changes after D-galactose induction (Figure 2(e)). In the D0 group, most of the mitochondria presented a short- and round-shaped morphology. Most of the mitochondria in D40 cells appeared as long, tubular, and sometimes branched or two-neighbored structures that spread throughout the entire cytoplasm. Notably, some of them were elongated and became the highly interconnected net-like mitochondria (indicated with red arrows). Thus, the structural features of the mitochondrial elongation thus represented abnormal mitochondrial dynamics in response to D-galactose. After resveratrol (100 *μ*M) treatment, we observed a decreased number of elongated mitochondria in H9c2 cells that displayed a tubular or ball-like structure without the connected-like part.

Meanwhile, we evaluated the effects of resveratrol on fission- or fusion-regulated proteins after D-galactose induction and found that resveratrol significantly upregulated Drp1 expressions in a dose-dependent manner (Figure 2(c)). We further analyzed whether resveratrol with or without D-galactose might play roles in cellular toxicity and mitochondrial damage. As shown in Figure 2(d), MTT and fluorescent staining experiments demonstrated that the cell and mitochondrial viabilities were not impaired by 24 hours of 100 *μ*M resveratrol treatment.

Taken together, these results indicated that Drp1 downregulation in response to D-galactose caused marked alterations in both mitochondrial length and distribution, revealing obvious elongation morphology. After resveratrol treatment, mitochondrial elongation was decreased, thus ameliorating the characteristic abnormalities in mitochondrial dynamics induced by D-galactose.

### 3.2. Resveratrol Alleviated Senescent-Like Cell Phenotypes in Response to D-Galactose Induction

**Figure 3:**
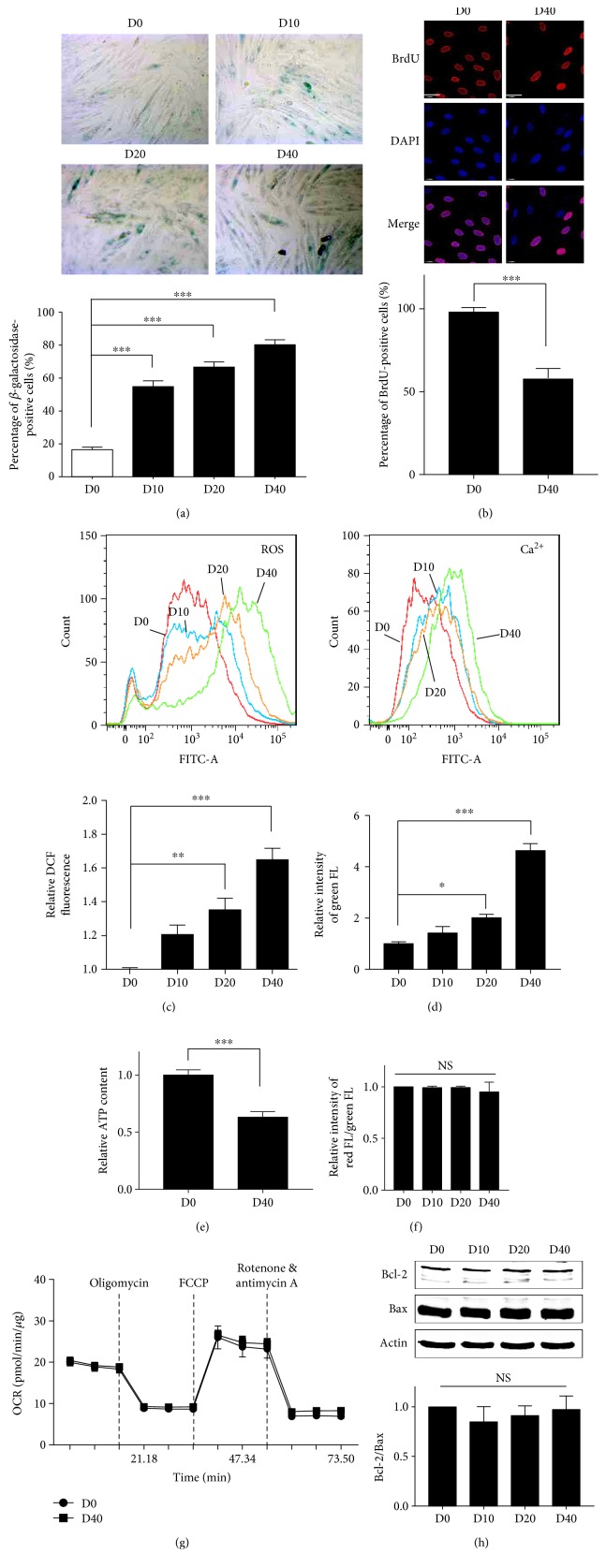
H9c2 cardiomyocytes displayed senescent-like phenotypes induced by D-galactose. (a) H9c2 cardiomyocytes were treated with D-galactose (D-Gal; 0, 10, 20, and 40 g/l). SA-*β*-Gal activity was detected using a microscope in a bright field with positive blue staining; original magnification, 100x; the percentage of positive staining was analyzed in five randomized fields. Data (*n* = 5) were shown as the mean ± SEM (^∗∗∗^*p* < 0.001). (b) BrdU activity was represented by BrdU (red) and DAPI (blue) double staining detected using a confocal microscope; objective magnification, 63x; scale bar represents 40 *μ*m. The percentage of positive staining was analyzed in five randomized fields. Data (*n* = 5) were shown as the mean ± SEM (^∗∗∗^*p* < 0.001). H9c2 cardiomyocytes were treated with D-galactose (D-Gal; 0, 10, 20, and 40 g/l). (c) ROS production was evaluated by using H_2_DCFDA probe staining, and (d) calcium concentration was evaluated by using Fluo-4 AM staining. Results were detected by flow cytometry. Data (*n* = 3) were shown as the mean ± SEM (^∗^*p* < 0.05, ^∗∗^*p* < 0.01, and ^∗∗∗^*p* < 0.001). (e) ATP content was detected by the luminescent detection assay. Data (*n* = 3) were shown as the mean ± SEM (^∗∗∗^*p* < 0.001). (f) Mitochondrial membrane potential (MMP) was detected using flow cytometry by JC-1 fluorescent dye staining. Data (*n* = 3) were shown as the mean ± SEM; NS: no significance (*p* > 0.05). (g) The H9c2 cardiomyocyte oxygen consumption rate was evaluated using the XFp Cell Mito Stress Test Kit and detected by the Seahorse Bioscience XFp Extracellular Flux Analyzer. (h) Bcl-2 and Bax protein expressions were analyzed by immunoblot analysis and calculated. Actin was represented as the loading control. Data (*n* = 3) were shown as the mean ± SEM; NS: no significance (*p* > 0.05).

Chronic D-galactose administration causes alterations that resembled natural aging in animals [27]. Here, we examined the senescence-related parameters in H9c2 cardiomyocytes in response to D-galactose. First, we performed the senescence-associated *β*-galactosidase (SA *β*-Gal) staining, a biomarker for senescent and aging cells. The percentage of positive blue staining in the D40 group increased to 83.16% compared with only 16.16% in the D0 group (Figure 3(a)). To further confirm a cellular senescent-like phenotype in response to D-galactose, the BrdU incorporation assay was performed to evaluate cell proliferation. Fluorescent images show that there was loss of BrdU/DAPI double staining in D40 cells compared with D0 cells. Quantification of cells with double staining showed 44.44% loss of BrdU staining, indicating a reduction in cardiomyocyte proliferation (Figure 3(b)). Furthermore, we used a fluorescent probe to detect the production of reactive oxygen species (ROS) and cellular calcium concentrations. Compared with D0 cells, the ROS productions in D20 and D40 cells were significantly increased (Figure 3(c), 1.71-fold increase in the D40 group compared with the D0 group). Meanwhile, calcium concentrations in the D20 and D40 were also significantly increased (Figure 3(d), 4.88-fold increase in the D40 group compared with the D0 group). Senescent cardiomyocytes are well-known to display reduced ATP production. Therefore, we examined the ATP content, and the result shows a 38.96% decrease in ATP content in D40 cells compared with D0 cells (Figure 3(e)).

We also wondered whether the elongated mitochondria were damaged in response to D-galactose induction. Therefore, we performed a mitochondrial membrane potential (MMP) assay to explore whether the mitochondria were depolarized which was induced by D-galactose. Interestingly, no significant change was found in the MMP in H9c2 cells induced by D-galactose (Figure 3(f)). In addition, we further detected mitochondrial respiration by testing the oxygen consumption rate. As shown in Figure 3(g), the H9c2 cellular oxygen consumption rate did not obviously change, suggesting that mitochondrial respiratory functionality was not impaired in D40 cells. To further evaluate proapoptotic protein expressions, we analyzed the expressions of Bcl-2 and Bax. No significant changes in the expression levels of Bcl-2 and Bax were detected in response to different doses of D-galactose (Figure 3(h)).

**Figure 4:**
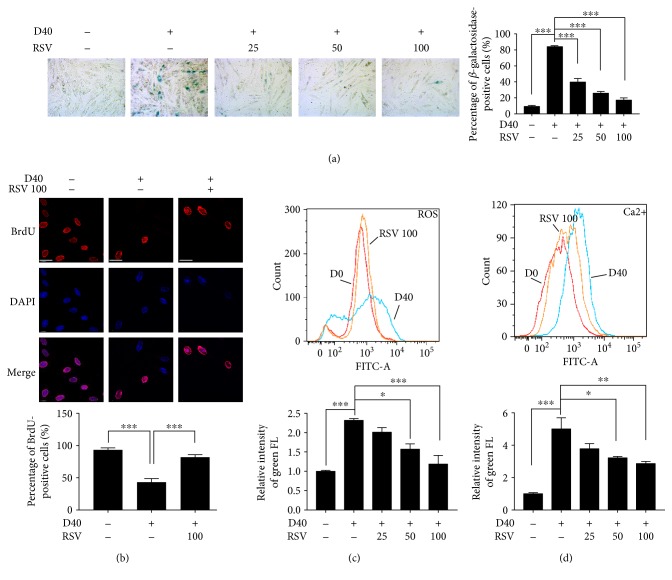
The effects of resveratrol on senescent phenotypes of H9c2 cardiomyocytes induced by D-galactose. (a) H9c2 cardiomyocytes were treated with resveratrol (RSV; 25, 50, and 100 *μ*M) for 12 hours after 48-hour induction of D-Gal (40 g/l). SA-*β*-Gal activity was detected using a microscope in a bright field with positive blue staining; original magnification, 100x; the percentage of positive staining was analyzed in five randomized fields. Data (*n* = 5) were shown as the mean ± SEM (^∗∗∗^*p* < 0.001). (b) BrdU activity was represented by BrdU (red) and DAPI (blue) double staining detected using confocal microscopy; objective magnification, 63x; scale bar represented 40 *μ*m. The percentage of positive staining was analyzed in five randomized fields. Data (*n* = 5) were shown as the mean ± SEM (^∗∗∗^*p* < 0.001). (c, d) H9c2 cardiomyocytes were treated with resveratrol (RSV; 25, 50, and 100 *μ*M) for 12 hours after 48-hour induction of D-Gal (40 g/l). ROS production was evaluated by using H2DCFDA probe staining, and calcium concentration was evaluated by using Fluo-4 AM staining. Results were detected by flow cytometry. Data (*n* = 3) were shown as the mean ± SEM (^∗^*p* < 0.05, ^∗∗^*p* < 0.01, and ^∗∗∗^*p* < 0.001).

We evaluated the effects of resveratrol on senescent-like phenotypes induced by D-galactose. As shown in Figure 4(a), resveratrol significantly and dose-dependently reduced D-galactose-induced SA *β*-Gal-positive staining and increased the percentage of BrdU/DAPI double staining (Figure 4(b)). Moreover, resveratrol significantly decreased the ROS production and calcium concentration in response to D-galactose, respectively, in a dose-dependent manner (Figures 4(c) and 4(d)).

Collectively, D-galactose induced cellular senescent-like phenotypes in H9c2 cardiomyocytes, including increased expression of a senescent biomarker, reduced cell proliferation, excessive ROS production, and calcium overloading. Regarding the functional aspect of mitochondria, no changes in the mitochondrial membrane potential or the respiration chain were observed. We also showed that the mitochondrial-mediated apoptotic signaling pathway was not activated. Importantly, resveratrol decreased SA *β*-Gal activity, increased cellular proliferation, and decreased ROS production and calcium overloading, thus ameliorating D-galactose-induced senescent-like phenotypes in H9c2 cardiomyocytes.

### 3.3. Resveratrol Reduced the Resistance of Elongated Mitochondria to CCCP-Induced Depolarization in Senescent-Like Cardiomyocytes

**Figure 5:**
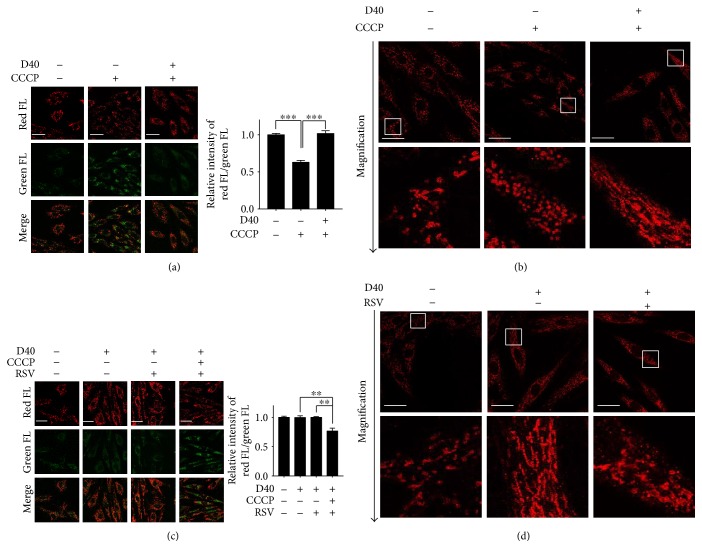
Resveratrol reduced the resistance of elongated mitochondria to CCCP-induced depolarization in H9c2 cells. (a, c) Mitochondrial membrane potential was detected using confocal microscopy by JC-1 fluorescent dye staining. Images were captured by confocal microscopy to show the variations of red and green FL; white scale bar represented 40 *μ*m. The ratio of red/green FL was calculated by the fluorescence microplate assay. Data (*n* = 3) were shown as the mean ± SEM (^∗∗^*p* < 0.01, ^∗∗∗^*p* < 0.001). (b, d) Mitochondrial morphology was detected using confocal microscopy (upper panels) by immunostaining with a TOM20 antibody (red); objective magnification, 63x; white scale bar represents 20 *μ*m. Magnified photographs showed a detail view of the area indicated in the upper panels.

The mitochondrial membrane potential (MMP) is considered a key sensor and/or effector of intracellular regulatory processes such as apoptosis, redox status, calcium homeostasis, and balance of mitochondrial fusion and fission. We next explored the relationship between mitochondrial depolarization induced by CCCP and mitochondrial elongation after D-galactose induction. We detected the MMP with JC-1 dye using fluorescence microscope- and microplate cytometry-based analyses. Incubating H9c2 cells with CCCP for 3 hours before JC-1 staining indeed increased the intensity of the green fluorescent signal (Figure 5(a), middle panels), indicating significant mitochondrial depolarization. However, we did not observe the CCCP-induced collapse of MMP in the 40 g/l D-galactose-treated H9c2 cells (D40, Figure 5(a), right panels). Using cytometry, we calculated the ratio of red FL to green FL, which represents contributions from both monomers and aggregates of JC-1. Consistent with the results shown in fluorescent images, we also found a decreased red FL/green FL ratio in H9c2 cells after CCCP stimuli, indicating significant loss of MMP. However, there was a significant attenuation of the ability of CCCP to trigger decreased MMP in D40 cells. Meanwhile, we also tested whether the distinct effect of CCCP on D40 cells occurred due to mitochondrial elongation in senescent-like cardiomyocytes. We examined mitochondrial morphological changes in cells exposed to CCCP by immunostaining with an anti-TOM20 antibody (red). Compared with the H9c2 cells without D-galactose induction, CCCP-treated cells contained a higher proportion of short and/or punctate mitochondria, revealing that CCCP leads to mitochondrial fragmentation. Interestingly, the effect of CCCP was not observed in cells in which mitochondrial elongation was induced by D-galactose, supporting our previous observation that normal or nonelongated mitochondrial morphology was required for CCCP-induced mitochondrial depolarization in cardiomyocytes (Figure 5(b)).

We then postulated that mitochondrial elongation might be an important determination in the MMP-mediated pathway. To determine whether the effects of resveratrol on mitochondrial elongation are involved in mitochondrial depolarization, we examined the MMP alterations and mitochondrial morphology in H9c2 cells after resveratrol treatment. As expected, resveratrol had no direct effect on the loss of MMP in D40 cells. Interestingly, in D40 plus CCCP cells, resveratrol significantly deceased the MMP, suggesting sensitized depolarization to mitochondrial decoupling (Figure 5(c)). Evaluation of mitochondrial morphology by immunostaining with TOM20 further validated the observation that the number of elongated mitochondria was reduced by resveratrol treatment compared with that in D40 cells (Figure 5(d)).

These data suggested that the resistance of mitochondria to CCCP-induced depolarization was likely due to abnormal elongation in D-galactose-induced senescent-like cardiomyocytes. After resveratrol treatment, the mitochondria were more susceptible to depolarization induced by CCCP, which probably resulted from mitochondrial elongation suppression.

### 3.4. Resveratrol Regulates Parkin/PINK1 Signaling upon Mitochondrial Depolarization in Senescent-Like Cardiomyocytes

**Figure 6:**
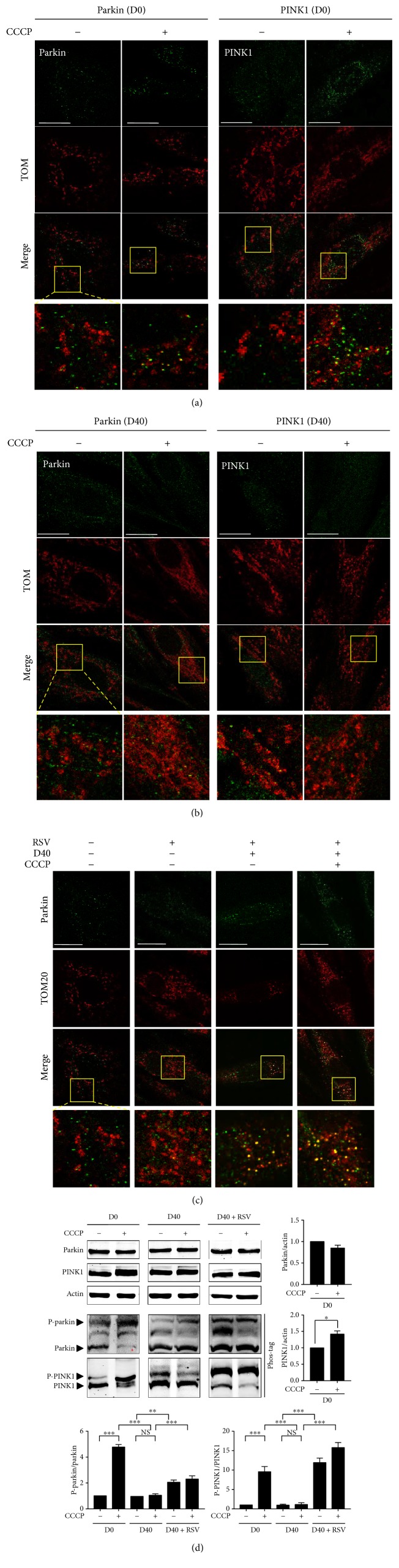
Resveratrol regulated the activity of parkin/PINK1 signaling upon mitochondrial depolarization in H9c2 cells in response to D-galactose induction. (a, b, c) Parkin and PINK1 expressions were detected using confocal microscopy by immunofluorescence analysis; objective magnification, 63x; scale bar represents 20 *μ*m. Colocalization of parkin (or PINK1) (green) and TOM20 (red) was shown in magnified images. The yellow fluorescence represented the overlap of red and green fluorescence. (d) Parkin and PINK1 protein expressions were detected by immunoblot analysis and calculated. Actin was represented as the loading control. Phosphorylation of parkin and PINK1, respectively, was detected by immunoblot analysis with phos-tag and incubated with anti-parkin and anti-PINK1 primary antibodies. The upper binds (slow migration binds) indicated by the black arrow showed phosphorylated proteins. Data (*n* = 3) were shown as the mean ± SEM (^∗^*p* < 0.05, ^∗∗^*p* < 0.01, and ^∗∗∗^*p* < 0.001).

First, we detected the parkin mitochondrial translocation by immunostaining with an anti-parkin antibody (green) and an anti-TOM20 antibody (red) using confocal microscopy in the H9c2 cells without D-galactose induction. After incubating the H9c2 cells with CCCP for 3 hours, we observed increased intensity of the yellow fluorescent signal in the merged image (Figure 6(a), second line from the left) compared with that in the cells without CCCP treatment (Figure 6(a), first line from the left). The yellow spots represented the overlaying of green and red fluorescence (FL), indicating that parkin translocated to mitochondria. Next, we examined the activation of PINK1 by immunostaining with an anti-PINK1 antibody (green) and an anti-TOM20 antibody (red). Compared with the H9c2 cells without CCCP treatment (Figure 6(a), second line from the right), the number of yellow dots was increased in the CCCP-treated H9c2 cells, indicating mitochondrial PINK1 translocation (Figure 6(a), first line from the right). We next accessed whether the CCCP-induced mitochondrial translocations of parkin and PINK1 were associated with their upregulations. We analyzed the protein levels of parkin and PINK1 after exposure to a CCCP stimulus. Immunoblot analysis showed that the total protein expression of parkin was not changed significantly (Figure 6(d)). Parkin phosphorylation is a critical step for its mitochondrial translocation in response to CCCP-induced mitochondrial depolarization. We therefore performed immunoblot analysis using an SDS-PAGE gel containing a phos-tag to investigate whether parkin was phosphorylated in H9c2 cells subjected to CCCP. In the phos-tag immunoblot, phosphorylated proteins appeared as slower migrated bands, and a significant increase in band intensity revealed that parkin was phosphorylated in the CCCP-treated cells. Interestingly, the total protein expression level of PINK1 was increased significantly which was induced by CCCP. Meanwhile, we observed an obvious slower PINK1 migration in its phos-tag immunoblot gel, which was potentially reflective of increased PINK1 phosphorylation. These results indicated that both parkin and PINK1 translocated to mitochondria in the presence of CCCP and suggested that higher parkin and PINK1 phosphorylation played a role in their activations.

To further address whether mitochondrial elongation affects the activations of parkin and PINK1 in senescent-like cardiomyocytes, we next investigated the mitochondrial translocation of parkin and PINK1 in the H9c2 cells induced by 40 g/l D-galactose (D40). In the merged images, we did not observe an obvious yellow fluorescent signal in D40 cells not treated with CCCP, indicating that parkin did not translocate to mitochondria (Figure 6(b), first line from the left). Interestingly, the CCCP-induced increase in yellow fluorescence was not detected in D40 cells, suggesting a significant attenuation in the ability of CCCP to trigger the mitochondrial translocation of parkin (Figure 6(b), second line from the left). Next, we analyzed the protein level of parkin induced by CCCP in the D40 cells. Compared with the D40 cells not treated with CCCP treatment, the total parkin expression level did not change significantly in the presence of CCCP (Figure 6(d)). Meanwhile, we also tested whether the CCCP-induced activation of PINK1 was exhibited only in D40 cells. As shown in Figure 6(b), D-galactose treatment did not induce the mitochondrial translocation of PINK1 (second line from the right). We did not observe PINK1 mitochondrial translocation in the D40 cells exposed to CCCP in the merged images (first line from the right). Immunoblot analysis of the PINK1 total protein level showed that its expression did not increase in response to CCCP treatment in the D40 cells. Furthermore, we tested whether the phosphorylation of parkin and PINK1 induced by CCCP was affected by D-galactose induction. In phos-tag immunoblots, we did not observe significant levels of phosphorylated parkin and PINK1 induced by CCCP in D40 cells. These data demonstrated that the CCCP-induced activation of parkin and PINK1 was decreased in the D40 cells, suggesting that mitochondrial depolarization induced by CCCP might be required for the activation of parkin and PINK1.

We next investigated the effects of resveratrol on the expressions of parkin and PINK1 in the D40 cells. First, resveratrol did not induce parkin mitochondrial translocation in D0 cells (Figure 6(c), second line from the left). Then, we examined parkin mitochondrial translocation in the D40 cells treated with resveratrol. In the merged images, we observed significantly increased yellow fluorescence signal intensity after 12 hours of resveratrol treatment (Figure 6(c), second line from the right). Interestingly, the number of colocalized yellow dots was increased in the presence of CCCP, suggesting that resveratrol increased CCCP-induced parkin mitochondrial translocation in the D40 cells (Figure 6(c), first line from the right). Moreover, after resveratrol treatment, the total expression levels of parkin and PINK1 were not significantly changed in response to CCCP induction in D40 cells. Therefore, we speculated whether parkin and PINK1 phosphorylation increased in response to resveratrol. As shown in Figure 6(d), phosphorylated parkin and PINK1 expressions were detected and showed a significant increase in D40 cells and D40 plus CCCP cells after resveratrol treatment, suggesting that parkin phosphorylation and PINK1 phosphorylation were implicated in parkin mitochondrial translocation induced by resveratrol in H9c2 cells.

Taken together, the CCCP-induced activation of parkin and PINK1 was suppressed in senescent-like H9c2 cardiomyocytes, most likely due to the resistance of elongated mitochondria to depolarization. After resveratrol treatment, parkin translocated to mitochondria due to the phosphorylation of parkin and PINK1.

### 3.5. Resveratrol Regulates LC3-Mediated Autophagy Induced by CCCP in Senescent-Like Cardiomyocytes

**Figure 7:**
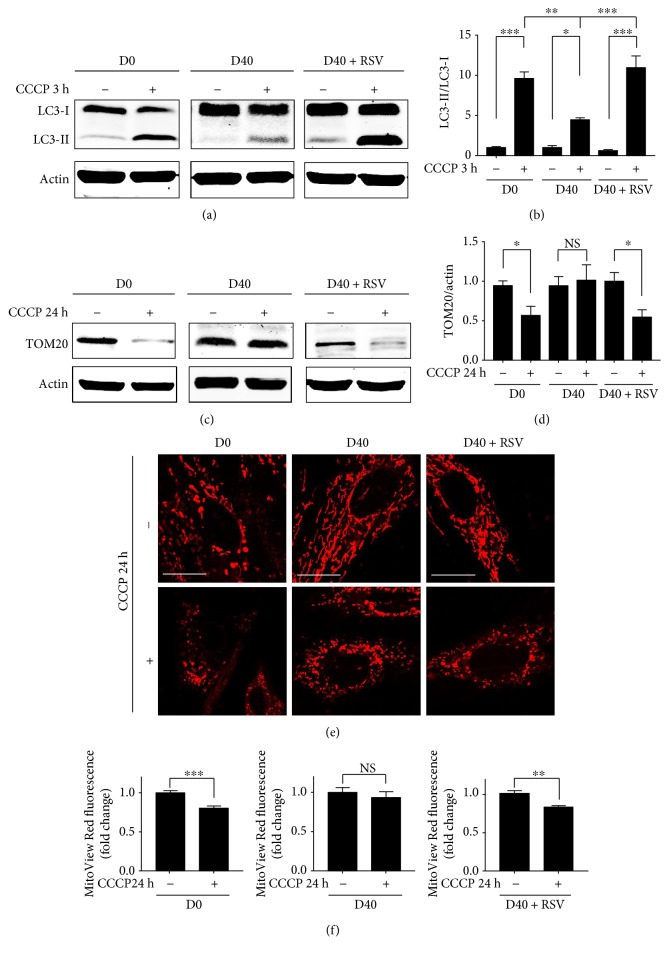
Resveratrol regulated the LC3-mediated autophagy induced by CCCP in D-galactose-induced H9c2 cells. (a) LC3-I and LC3-II expressions in H9c2 cells treated with CCCP (80 *μ*M) for 3 hours were detected by immunoblot analysis. Actin was represented as the loading control. (b) The relative values of LC3-II/LC3-I expressions were calculated. Data (*n* = 3) were shown as the mean ± SEM (^∗^*p* < 0.05, ^∗∗^*p* < 0.01, and ^∗∗∗^*p* < 0.001). (c) TOM20 expressions in H9c2 cells treated with CCCP (80 *μ*M) for 24 hours were detected by immunoblot analysis. Actin was represented as the loading control. (d) The relative values of TOM20 expressions were normalized to actin. Data (*n* = 3) were shown as the mean ± SEM (^∗^*p* < 0.05); NS: no significance (*p* > 0.05). (e) H9c2 cardiomyocytes were treated with CCCP (80 *μ*M) for 24 hours. Mitochondrial morphology was detected using confocal microscopy by MitoView Red staining; objective magnification, 63x; white scale bar represented 20 *μ*m. (f) Quantification of MitoView Red staining dye was assessed by the fluorescence microplate assay. Data (*n* = 3) were shown as the mean ± SEM (^∗∗^*p* < 0.01, ^∗∗∗^*p* < 0.001); NS: no significance (*p* > 0.05).

As an E3 ubiquitin ligase, parkin is targeted to damaged mitochondria and mediates its selective removal via damaged protein degradation by autophagosomes or lysosome. We therefore examined the LC3-II/LC3-I conversion induced by CCCP in the H9c2 cells and calculated the ratio of LC3-II to LC3-I by immunoblot analysis.  Figure 7(a) shows that CCCP (80 *μ*M) treatment for 3 hours significantly increased the expression of LC3-II in D0 cells (left panels). In D40 cells, we observed that the increased expression of LC3-II after CCCP treatment was significantly lower than that in D0 cells (middle panels). As shown in Figure 7(b), the ratio of LC3-II to LC3-I was increased by approximately 10-fold and 5-fold in D0 and D40 cells, respectively, in the presence of CCCP. Resveratrol treatment significantly increased the expression of LC3-II (Figure 7(a), right panels), and the ratio of LC3-II increased more than 10-fold in RSV plus CCCP-treated D40 cells. This result implied that the suppression of parkin and PINK1 contributed to the reduced activity of LC3-II in D-galactose-treated H9c2 cardiomyocytes. Resveratrol enhanced the ability of CCCPs to upregulate LC3-II expression, suggesting that it affected LC3-mediated autophagy.

We also investigated whether RSV affected the TOM20-labeled mitochondria in H9c2 cells treated with CCCP. After 24 hours of treatment with CCCP, the expression of TOM20 was downregulated significantly in the D0 cells (Figure 7(c), left panels). The relative expression intensity was calculated, and the results in Figure 7(d) show that the expression of TOM20 was downregulated by approximately 50% in D0 cells treated with CCCP for 24 hours. Meanwhile, in the D40 cells, the expression of TOM20 did not change significantly after CCCP induction (Figure 7(c), middle panels). Interestingly, the expression of TOM20 was significantly downregulated by CCCP in the D40 cells treated with resveratrol (Figure 7(c), right panels), indicating that the effect of resveratrol is probably involved in the mitochondrial autophagy process that was disturbed by D-galactose in H9c2 cells. To confirm the loss of mitochondrial content, we visualized the mitochondria using fluorescence staining by MitoView Red and calculated the fluorescence intensity. In D0 cells, the morphology of mitochondria significantly changed to very short, fragmented spots after CCCP induction. The mitochondrial distribution was observed in parts of the cytoplasm, although some disappeared (Figure 7(e), left panels). The intensity of the mitochondrial fluorescence staining dye was significantly decreased, indicating the loss of mitochondrial content (Figure 7(f), left panel). Although the mitochondria were obviously fragmented in D40 plus CCCP cells, no significant loss of mitochondrial content could be detected (Figures 7(e) and 7(f), middle panels). Interestingly, after resveratrol treatment, the mitochondrial content was significantly decreased in D40 plus CCCP cells (Figures 7(e) and 7(f), right panels).

## 4. Discussion

In our study, mitochondrial dynamic abnormalities were found and most mitochondria became tubular or thread-like in response to D-galactose induction. The major regulators of mitochondrial morphology were fission and fusion proteins, suggesting that mitochondrial elongation is probably caused by the imbalance of fission and fusion [28]. Immunoblot analysis showed that Drp1 might be the major factor contributing to mitochondrial elongation induced by D-galactose. Mdivi-1 was a mitochondrial fission inhibitor that induced mitochondrial elongation by inhibiting fission machinery [12]. Our study showed that the mitochondria were elongated and the expression of Drp1 was downregulated in response to Mdivi-1 induction. Compared with the cells induced by Mdivi-1, D-galactose-treated cells showed similar mitochondrial morphology and Drp1 expression, suggesting that mitochondrial elongation induced by D-galactose was partially due to defective fission. Regarding fusion proteins, 40 g/l D-galactose induction did not alter the expressions of Mfn1, Mfn2, or OPA1. A study by Neuspiel et al. showed that mitofusin 2 (Mfn2) acted as a signaling GTPase to induce mitochondrial fusion and protected against permeability transition [29], suggesting that Mfn2-mediated mitochondrial fusion enhances the endurance of cardiomyocytes to depolarization. However, specific Mfn2 knockout produced enlarged mitochondria that exhibited impaired respiratory function [30]. In addition, independent of Mfn2 promoting fusion, this protein played a critical role in culling damaged mitochondria [9]. Therefore, the roles of Mfn2 in the formation of enlarged mitochondria are complex and not fully elucidated. In our study, Drp1-mediated fission played a major role in mitochondrial elongation in response to D-galactose induction. Resveratrol significantly decreased the mitochondrial elongation in H9c2 cells, thus attenuating the characteristic mitochondrial dynamic abnormalities induced by D-galactose.

Previous studies have evaluated several parameters to show the effects of D-galactose on cellular senescence, such as advanced glycation end products (AGEs), superoxide dismutase (SOD), and telomeres [13, 31–33]. Because cellular senescence is a very complex process that is not fully elucidated, here, we analyzed the senescent-like phenotypes in H9c2 cardiomyocytes in response to D-galactose by evaluating senescence-associated *β*-galactosidase activity, a biomarker for cellular senescence or organ aging [34]. For cellular senescence and proliferation [35], we observed reduced cell proliferation by the BrdU incorporation assay. Furthermore, several other metabolism disorder indicators, such as intracellular calcium overloading, enhanced ROS production enhancement, and decreased levels of ATP production, were found in H9c2 cardiomyocytes induced by D-galactose. Taken together, D-galactose induction increased the activity of senescence-associated *β*-galactosidase and reduced cellular proliferation, thus inducing senescent-like phenotypes in H9c2 cardiomyocytes. Moreover, H9c2 cells displayed excessive ROS production and intracellular calcium overloading induced by D-galactose. Aging cardiomyocytes produced damaged and enlarged mitochondria that exhibit excessive ROS and mutated respiratory function genes and proteins, which make them more difficult to selectively remove than smaller mitochondria [6]. Therefore, we further evaluated the mitochondrial membrane potential and respiration in response to D-galactose. D-galactose induction did not change the MMP and oxygen consumption rate, implying that the mitochondrial function was not impaired. Further analysis of expressions of Bcl-2 and Bax suggested that the mitochondrial-mediated apoptotic signaling pathway was not activated. Further evaluations of resveratrol on senescent-like phenotypes induced by D-galactose showed that resveratrol significantly reduced SA *β*-Gal-positive staining and increased the percentage of BrdU/DAPI double-positive staining. In addition, resveratrol significantly decreased the ROS production and calcium concentration in response to D-galactose, respectively, in a dose-dependent manner.

Mitochondrial elongation with abnormal dynamics was shown to alter small molecular activation and/or intracellular pathways, including the balance of fusion and fission and the selective removal of damaged mitochondria, probably contributing to the reduction of IPC in the development of cardiac aging. Drp1-mediated mitochondrial fission is considered a potential upstream effector for subsequent selective removal of mitochondria [20]. Evidence showed that decreased Drp1 expression damaged mitochondria, thus increasing cardiomyocyte apoptosis and suppressing glucose deprivation-induced autophagosome formation and autophagic flux. Ikeda et al. applied mitochondrial-targeted Keima fluorescence to monitor the maturation of autophagosomes to lysosomes by detecting excitation spectra peaking in acidic pH conditions. The CCCP-induced positive staining of Keima on mitochondria was not detected in cardiomyocytes transduced with Ad-shDrp1, suggesting that Drp1 was necessary for mitochondrial autophagic removal. More direct evidence that Drp1 downregulation decreased the number of autophagosomes and autophagosomes containing mitochondria in response to glucose deprivation was detected by electron microscopy. Moreover, the authors also investigated the autophagic flux in Drp1-CKO mice. In Drp1-CKO mice, the activity of LC3-II was suppressed and the autophagic flux was present under both physical and chemical stimuli in the heart [19]. This result leads to the hypothesis that Drp1-mediated fission machinery probably dominates the separation of damaged mitochondrial components. Drp1-mediated mitochondrial fission was shown to segregate damaged components for selective elimination and to recruit parkin to mitochondria to maintain their integrity. Suppression of Drp1 reduced the mitochondrial translocation of parkin [20]. Parkin, an E3 ubiquitin ligase, was required in this process to promote Drp1-dependent mitochondrial fragmentation [36]. Furthermore, PINK1, a serine-threonine kinase that collaborates with parkin to regulate mitochondrial programmed clearance, was related to Drp1-mediated mitochondrial fission [37]. A prevalent signaling pathway implicated in selective mitochondrial removal was determined to be mediated by the parkin and PINK1 proteins [38]. Mitochondrial depolarization activated the outer membrane localization of PINK1 and drove cytosolic parkin recruitment [39, 40]. To investigate whether Drp1-mediated mitochondrial elongation in senescent-like cardiomyocytes suppressed selective mitochondrial removal, CCCP was used to induce mitochondrial depolarization and parkin and PINK1 activation.

CCCP is a conventional compound that causes mitochondrial depolarization and induces parkin mitochondrial translocation. In our study, no CCCP-induced mitochondrial depolarization was found in H9c2 cells in response to D-galactose. Interestingly, the mitochondria retained their elongated morphology in the presence of CCCP, indicating that elongated mitochondria exhibited resistance to CCCP-induced mitochondrial depolarization. Additionally, resveratrol ameliorated abnormal mitochondrial elongation by upregulating the expression of Drp1 in D-galactose-treated cardiomyocytes. Therefore, resveratrol probably could enhance the ability of CCCP to induce mitochondrial depolarization. After resveratrol treatment, the mitochondria were depolarized which was induced by CCCP in D-galactose-treated H9c2 cardiomyocytes. Next, we explored the activation of parkin and PINK1 to determine whether the resistance of elongated mitochondria to CCCP-induced depolarization would affect parkin mitochondrial translocation. Our results showed that parkin translocated to mitochondria in response to CCCP induction. However, we did not observe significant parkin mitochondrial translocation in D-galactose-treated cells that were resistant to CCCP-induced depolarization. Meanwhile, the results of PINK1 expression analysis by immunostaining showed that its activation was reduced, suggesting that the defective parkin and PINK1 mitochondrial translocations were implicated in the resistance of D-galactose-treated cells to CCCP-induced mitochondrial depolarization. Furthermore, PINK1-mediated parkin phosphorylation and PINK1 autophosphorylation were essential for the mitochondrial translocation of parkin upon mitochondrial depolarization [41, 42]. To examine the phosphorylation of parkin and PINK1, we applied SDS-PAGE gels containing a phos-tag to specifically separate the phosphorylated proteins for detection using immunoblot analysis. The phos-tag gel showed that the phosphorylation levels of parkin and PINK1 were enhanced in response to CCCP induction. However, the increased levels of phosphorylation were not observed in the cells induced by D-galactose, indicating that the phosphorylation of parkin by PINK1 was reduced and suggesting that Drp1-mediated mitochondrial elongation attenuated the activations of parkin and PINK1. Furthermore, CCCP-induced LC3-II protein upregulation and loss of mitochondrial content were suppressed in senescent-like cardiomyocytes, possibly, due to the attenuated activation of parkin.

Mutations in genes encoding the parkin and PINK1 proteins were first identified in the pathogenesis of Parkinson's diseases and determined to be involved in the underlying mechanisms of several other major neurodegenerative diseases [43, 44]. Decreased mitochondrial membrane potential and impaired respiratory chain were found in parkin-mutant patients and PINK1-mutant patients [45, 46]. Defective dysfunctional mitochondrial removal was revealed in the progress of neurodegenerative diseases with the absence of functional parkin and PINK1 [47, 48]. The potential mechanism elucidated by researchers was that the functional interaction of parkin and PINK1 displayed the clearance of dysfunctional mitochondria [49]. Despite the epidemiological association between Parkinson's diseases and heart failure, the indispensable roles of parkin and PINK1 for normal heart function have come under intense attack in the past decade. Evidence showed that deficient parkin protein levels in mice enhanced their sensitivity to cardiac infarction and larger infarct sizes [50]. Decreased PINK1 protein levels led to impaired mitochondrial function and redox homeostasis in cardiac dysfunction [51]. Moreover, the translocation of parkin to damaged mitochondria was disturbed in aged hearts. The overexpression of parkin ameliorated cardiac aging and maintained mitochondrial integrity [52]. In addition, Huang et al.'s study showed that parkin was involved in the cardioprotection of ischemic preconditioning and short-term hypoxia increased parkin mitochondrial translocation in cardiomyocytes [53]. These studies indicated that parkin plays a critical role in mitochondrial integrity in aging hearts, and defective parkin mitochondrial translocation might be a possible reason for the loss or attenuation of cardiac ischemic preconditioning.

**Figure 8:**
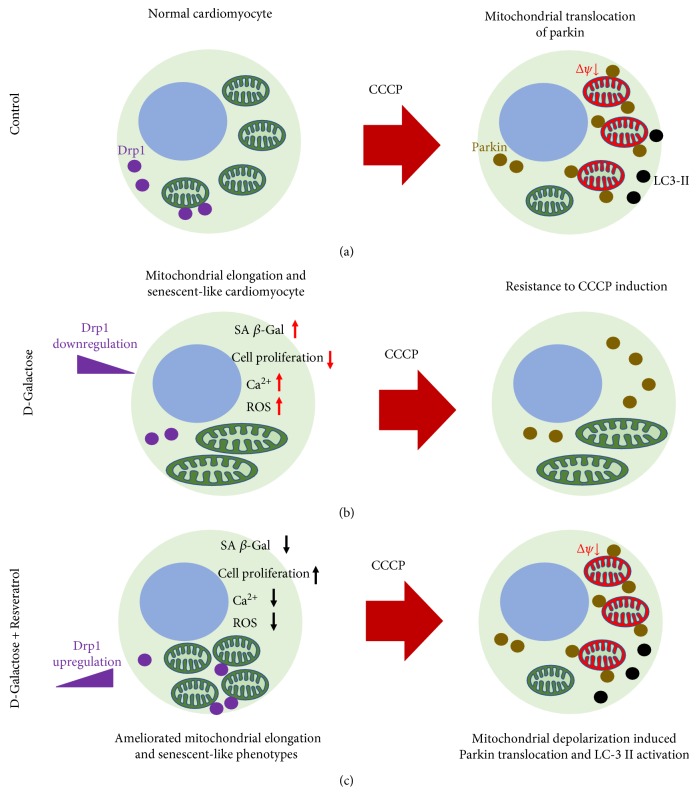
The schematic representation proposed the effect of resveratrol on mitochondrial elongation in D-galactose-induced cardiomyocytes. (a) Control, (b) D-galactose, and (c) D-galactose + resveratrol.

Resveratrol is a polyphenol compound that functions in age-related disorders [54]. Resveratrol can reportedly improve cardiac function in failing hearts by activating the SIRT1 protein level and improving AMPK expression [55]. The effect of resveratrol on mitochondria has been studied for years. Resveratrol was shown to exhibit multiple effects on mitochondrial mass, mtDNA content, and upregulation of the biogenetic factor of PGC-1*α*, supporting the role of resveratrol against myocardial ischemia/reperfusion injury by decreasing ROS generation and inhibiting mPTP opening [56]. Meanwhile, resveratrol has been shown to exhibit protective effects against oxidative stress by modulating mitochondrial biogenesis in an SIRT3-dependent manner after activating AMPK-PGC-1*α*-ERR*α* signaling [57]. Notably, our study is the first to investigate the effects of resveratrol on mitochondrial dynamics. Morphological studies of mitochondria by live cell staining and ultrastructural detection demonstrated that resveratrol ameliorated mitochondrial elongation by upregulating Drp1 expression. Therefore, our study provided a new viewpoint in the effects of resveratrol on mitochondrial dynamic imbalance, suggesting that the potential mechanisms of resveratrol antiaging properties are probably related to mitochondrial dynamics (Figure 8). Meanwhile, resveratrol increased the level of mitochondrial depolarization, perhaps by ameliorating mitochondrial elongation. For decades, few studies have explored the effects of resveratrol on parkin and PINK1 protein expressions and the signaling pathways in which they are involved. Recently, Das et al. showed that raised resveratrol exhibited the potential effects on a Sirt1-Sirt3-Foxo3-PINK1-PARKIN signaling network during ischemia/reperfusion injury [58]. Although their study provided promising insight into the effective mechanisms of resveratrol that involve parkin and PINK1, fully understanding the roles of resveratrol on the functional activation of parkin and PINK1 in different pathological processes still needs to be accomplished. Promisingly, our study showed that resveratrol significantly induced parkin mitochondrial translocation in senescent-like H9c2 cardiomyocytes by regulating the phosphorylation of parkin and PINK1. Furthermore, resveratrol upregulated the expression of LC3-II and decreased the mitochondrial content in D-galactose-induced cells, suggesting that it affected LC3-mediated autophagy.

## 5. Conclusion

In summary, our study characterized mitochondrial dynamic disorders in H9c2 cardiomyocytes that presented cellular senescent-like phenotypes in response to D-galactose induction. We observed marked mitochondrial elongation by Drp1-mediated fission disorders. Interestingly, D-galactose-treated cardiomyocytes showed resistance to mitochondrial depolarization, and their mitochondrial morphology exhibited elongation in response to CCCP induction. Importantly, the natural polyphenol compound resveratrol ameliorated mitochondrial elongation by enhancing Drp1 expression and increasing the level of mitochondrial depolarization, suggesting that mitochondrial elongation is partly implicated in depolarization resistance. Furthermore, the reduced activations of parkin and PINK1, including mitochondrial translocation and protein phosphorylation, in response to CCCP induction were found in senescent-like cardiomyocytes. Finally, resveratrol upregulated the mitochondrial translocation of parkin, activated the phosphorylation of parkin and PINK1, and upregulated the LC3-II activity, providing a new pharmacological approach for further studies of the mechanisms of Drp1 and parkin-PINK1 signaling.

## Abbreviations

IPC: Ischemic preconditioning
ATP: Adenosine triphosphate
K_ATP:_: ATP-sensitive potassium channels
MMP: Mitochondrial membrane potential
ROS: Reactive oxygen species
Mfn1: Mitofusin 1
Mfn2: Mitofusin 2
OPA1: Optic atrophy 1
Drp1: Dynamin-related protein-1
Mdivi-1: Mitochondrial division inhibitor 1
PINK1: PTEN-inducible kinase 1
SIRT1: Sirtuin 1
Bcl-2: B-cell lymphoma 2
Bax: Bcl-2-associated X
RSV: Resveratrol
CCCP: Carbonyl cyanide 3-chlorophenylhydrazone
BrdU: Bromodeoxyuridine
LC3: Microtubule-associated protein 1A/1B light chain 3.
